# The Corkscrew Technique for Removing a Fibular Strut Allograft From the Proximal Humerus

**DOI:** 10.7759/cureus.23233

**Published:** 2022-03-16

**Authors:** Brian Skura, Matthew T Glazier, Hayden B Schuette, Braden J Passias, Iou-Ren Chang, John Verre, Sanjay Mehta, Benjamin C Taylor

**Affiliations:** 1 Orthopedic Surgery, OhioHealth, Columbus, USA; 2 Orthopedic Surgery, OhioHealth Grant Medical Center, Columbus, USA; 3 Orthopedic Surgery, OhioHealth Doctors Hospital, Columbus, USA; 4 Orthopedic Surgery, Orthopaedic Associates of Wausau, Wausau, USA; 5 Orthopedic Trauma, OhioHealth Grant Medical Center, Columbus, USA

**Keywords:** lateral locking plate failure, corkscrew, revision shoulder arthroplasty, proximal humerus fracture, fibular strut allograft

## Abstract

A fibular strut allograft is a reliable option for augmentation in open reduction internal fixation (ORIF) of proximal humerus fractures, but techniques to remove a fibular strut during revision shoulder arthroplasty are limited. Currently published techniques on extracting fibular strut grafts from humeral shafts include using a Midas burr, flexible osteotomes, humeral shaft osteotomy, and reaming. To our knowledge there has not been a technique that uses a corkscrew to remove the fibular strut from the proximal humerus in preparation for revision shoulder arthroplasty. This is a case report and description of a simple and reproducible technique that can be implemented in the setting of conversion from a proximal humerus lateral locking plate with fibular strut allograft to shoulder arthroplasty.

## Introduction

Proximal humerus fractures are the third most common fracture in adults [1]. They comprise 4-5% of all fractures and most commonly occur in adults between the ages of 45-64 years old after a fall from standing height [2]. The incidence of proximal humerus fractures continues to rise with the aging population and they are predicted to be the cause of approximately 275,000 emergency department visits in 2030 [2]. Indications to surgically fix acute proximal humerus fractures are based on displacement, stability of the fracture fragments, bone quality, and patient’s physiologic age [3,4]. There are various techniques and treatment options available for fixation of these fractures.

The most widely used operative fixation method for most three- and four-part proximal humerus fractures in the young adult patient (<65 years old) is open reduction and internal fixation (ORIF) [3,4]. The advantage of ORIF with a lateral locking plate is preservation of native bone and restoration of anatomic joint congruity. Though studies have shown good results with the use of locking plate fixations, fracture patterns that are highly comminuted, especially at the calcar, present unique challenges and increase complications [5-10]. One strategy being implemented to decrease complication rates with lateral locking plate is adding a fibular strut allograft to provide endosteal augmentation. Studies have shown adding a fibular strut does improve outcomes and decreases complication rates [11-17]. However, when a fibular strut fails, this leaves the patient with limited options and poses the surgeon with a difficult decision.

Complications and failures after implementing a fibular strut present a unique challenge for revision surgery because most shoulder replacement options have stemmed humeral implants. This leaves the surgeon with the task of removing the previously placed endosteal strut which can be difficult due to impaction and bony ingrowth. There is a paucity within the literature for techniques and strategies for safely removing the strut without damaging the surrounding bone [18,19].

We present a case in which ORIF with a lateral locking plate and fibular strut allograft failed due to secondary screw cut-out and was revised to a reverse shoulder total arthroplasty (RTSA). Here we describe a novel technique using a simple handheld corkscrew to aid in removal of a fibular strut.

## Case presentation

**Figure 1:**
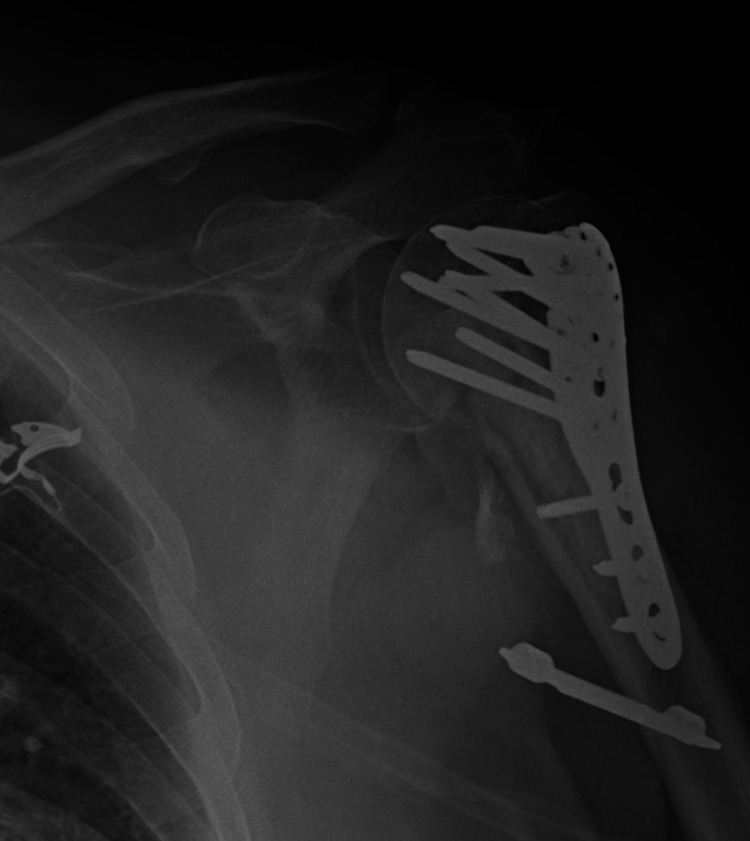
Postoperative anteroposterior (AP) radiograph of the left shoulder status post open reduction and internal fixation (ORIF) with fibular strut augmentation.

The patient is a 67-year-old male that initially presented to our urban Level 1 Trauma Center with a left proximal humerus fracture after a fall down a hill. Due to the poor bone stock, medial calcar comminution and significant varus collapse of the fracture, the patient had an allograft fibular strut placed as an augment with a DepuySynthes (Raynham, MA, USA) proximal humerus locking plate (Figure 1). 

**Figure 2:**
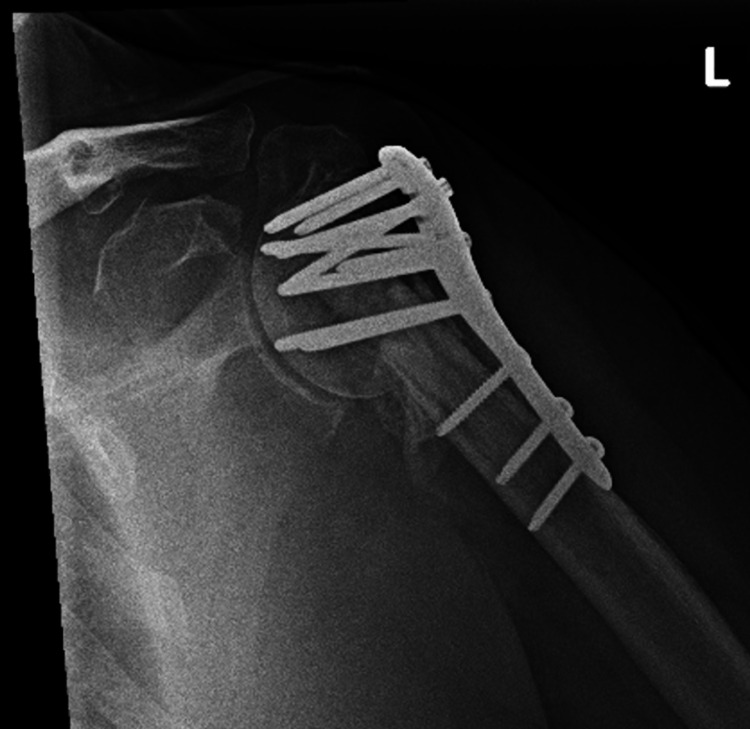
Anteroposterior (AP) radiograph of the left shoulder with varus collapse and failure of fibular strut augmentation.

Approximately 11 months from his revision ORIF the patient was having pain and impingement symptoms in his shoulder. On radiographs obtained at follow-up, it was noted the patient had varus collapse and failure of the fracture (Figure 2).

Due to pain and fracture displacement, the patient was then taken to the operating room for removal of hardware and fibular strut allograft with conversion to a reverse total shoulder arthroplasty during the same procedure. There were no complications noted using the corkscrew during removal of the fibular strut allograft.

At 18-month follow-up the patient regained 90 degrees of flexion and abduction, full internal rotation, and 15 degrees of external rotation in the affected shoulder. The patient has no pain with range of motion of the shoulder. His strength is preserved, and he is able to perform all activities of daily living.

Pre-operative imaging

**Figure 3:**
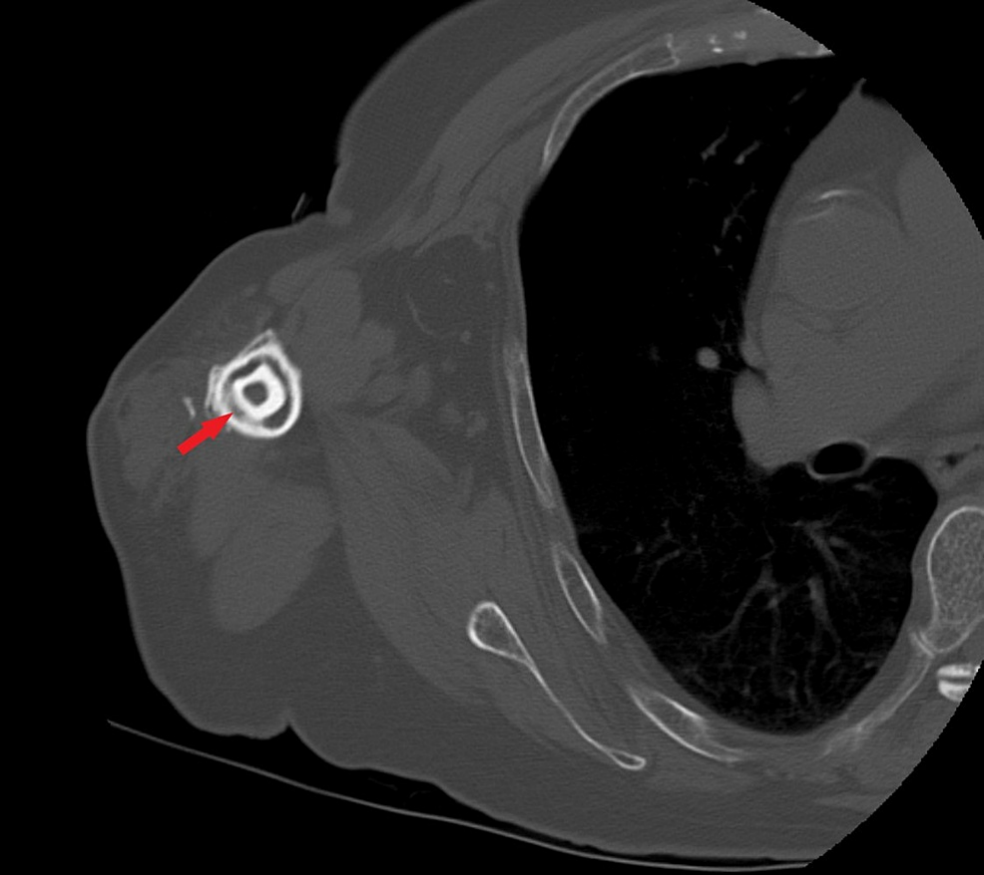
Axial cut of CT scan of the proximal humerus demonstrating osseous integration of fibular strut into the humeral canal (red arrow).

Standard pre-operative radiographs, anteroposterior (AP), Grashey and scapular Y, are taken in the clinic. Radiographs after ORIF should be scrutinized for fracture healing, hardware failure, collapse, and avascular necrosis (AVN) of the head. Advanced imaging such as computerized tomography (CT) scan can be useful to validate osseous integration and bony ingrowth between the fibular strut and cortex (Figure 3) [20].

**Figure 4:**
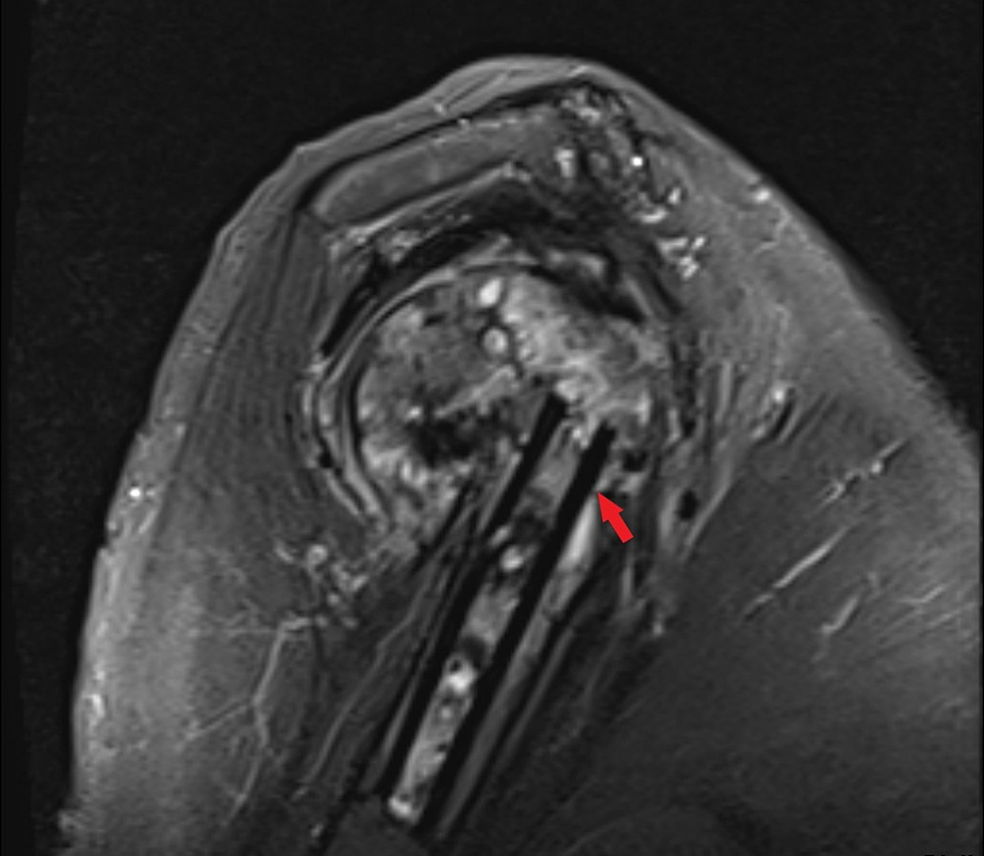
T2-weighted MRI of the proximal humerus demonstrating minimal osseous integration (red arrow).

Lastly, magnetic resonance imaging (MRI) is another advanced imaging technique that has been shown to be useful for evaluating the extent of augmentation integration. Lazaro et al. describe a scoring system of either 0, 1 or 2 indicating none, partial or complete integration into the cortical bone [21]. The more incorporation of the strut into the cortex the more the outer line of the cortex gets irregular and hyperintense along the whole strut allograft (Figure 4).

Obtaining advanced imaging can be difficult due to insurance approval, timing, and if there is hardware in place then artifact may obliterate any useful findings. Radiographic findings must correlate with clinical examination to ensure the best treatment plan is chosen for each patient.

Indications

There are only a few cited indications for conversion from ORIF of the proximal humerus to RTSA. The primary indication for conversion to RTSA after failed ORIF is pain, low patient satisfaction and functional limitation [22,23]. Failure of ORIF is subjective but can be generally defined as hardware failure such as screw cut-out or penetration, malunion, nonunion, or AVN of the humeral head.

Contraindications

Contraindications for conversion of RTSA from failed ORIF of the proximal humerus are the same as primary RTSA: deltoid dysfunction, axillary nerve palsy, active infection, neuropathic joint and glenoid insufficiency [24]. Deltoid function is critical for optimal results following RTSA and can be compromised with axillary nerve injury. History and physical examination as well as details of any complication from prior shoulder surgery, such as axillary nerve injury, should be investigated. If there is concern for a non-functioning deltoid, electromyography (EMG) should be performed to test the axillary nerve. EMG results can elucidate potential for recovery and whether there is partial or complete palsy. In cases of partial injury, the surgeon has the option to wait until the nerve recovers. However, if there is complete axillary nerve palsy, there are a limited number of options and other avenues such as shoulder fusion should be discussed with the patient.

Active infection must be ruled out prior to any revision surgery. Baseline inflammatory markers, C-reactive protein (CRP), erythrocyte sedimentation rate (ESR) and white blood cell count (WBC) must be checked. If there is elevation in these markers a glenohumeral aspiration should be the next step and synovial fluid analysis, cell count, cultures, Gram stain and crystals should be checked. Cultibacterium acnes (P. acnes) cultures can take five to 14 days in an anaerobic atmosphere before growth detection [25]. Therefore, revision surgery should be delayed until results of final cultures.

Prior to surgery the patient should be in overall good health and require pre-operative medical evaluation to ensure the patient is medically optimized. Chronic obstructive pulmonary disease (COPD), elevated hemoglobin A1c, cardiac disease, renal disease and obesity have all been linked to increased morbidity and mortality following major surgery [26]. A discussion with a patient with multiple medical comorbidities about goals of surgery and increased risk is vital for both patient and surgeon satisfaction and safety.

Technique overview

The patient was placed in a supine position with the left upper extremity prepped and draped in usual sterile fashion. The previous deltopectoral incision and interval were utilized. The arm was internally rotated, and the lateral locking plate and screws were identified. Once all the hardware was removed the glenohumeral joint was exposed and the humeral head was resected. Part of the fibular strut was attached to the head that was removed with an osteotome; however, there was still a significant portion of fibular strut within the humeral canal.

Corkscrew technique

Depending on the length of time the fibular strut has been in place, there will be varying amounts of osteointegration between the fibular strut and humeral interface. Pre-operative imaging, which is detailed earlier in this article, can aid in determining the difficulty of removing the strut. Begin with the smallest flexible osteotome in a circumferential manner to work around the graft to loosen the fibula-humeral bony interface. Care must be taken to minimize plunging the osteotomes down the shaft to avoid humeral cortex penetration. Once it is deemed the fibular strut is adequately separated from the humeral shaft, attention is turned to removing the strut itself.

**Figure 5:**
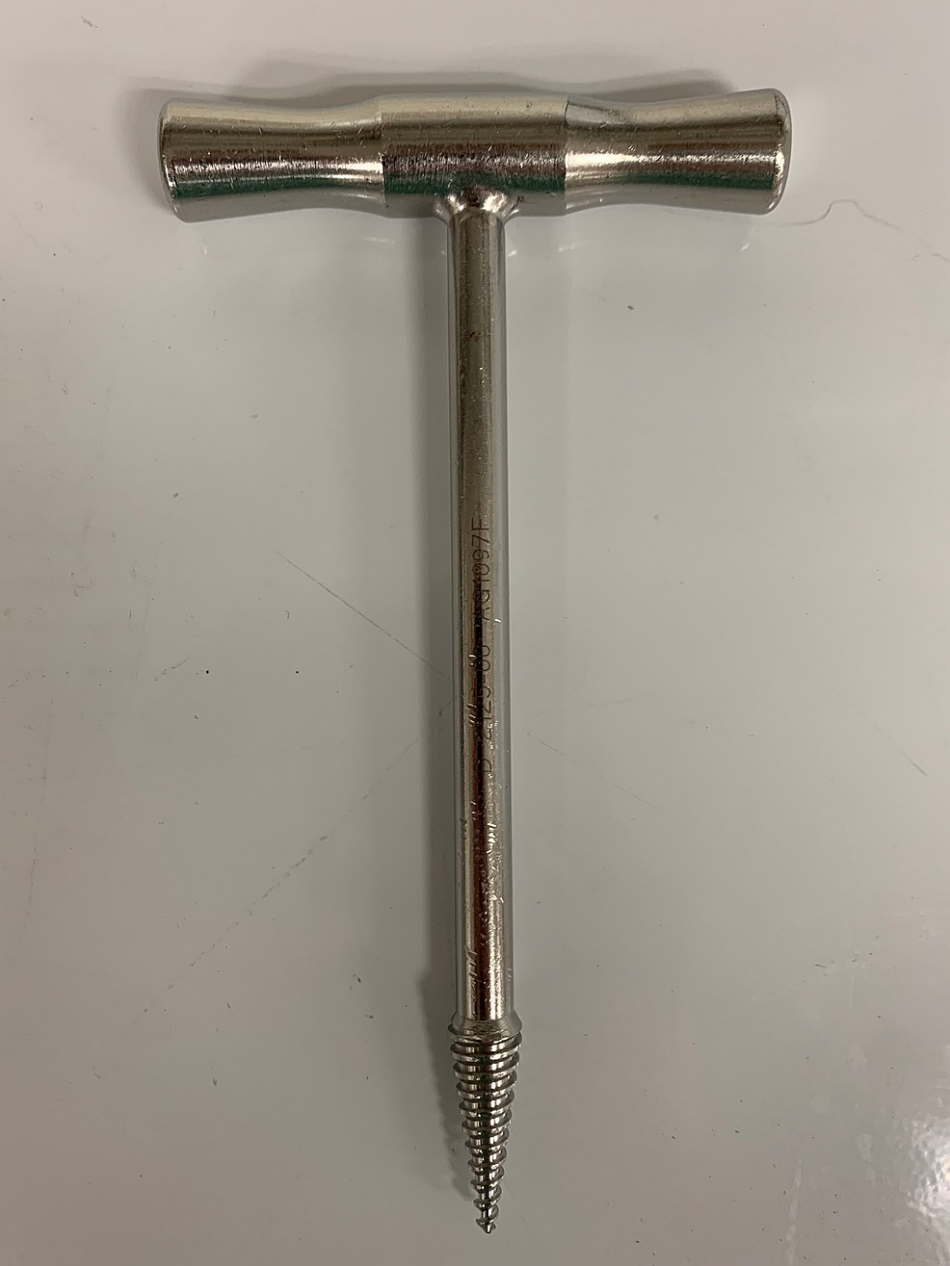
Handheld corkscrew

**Figure 6:**
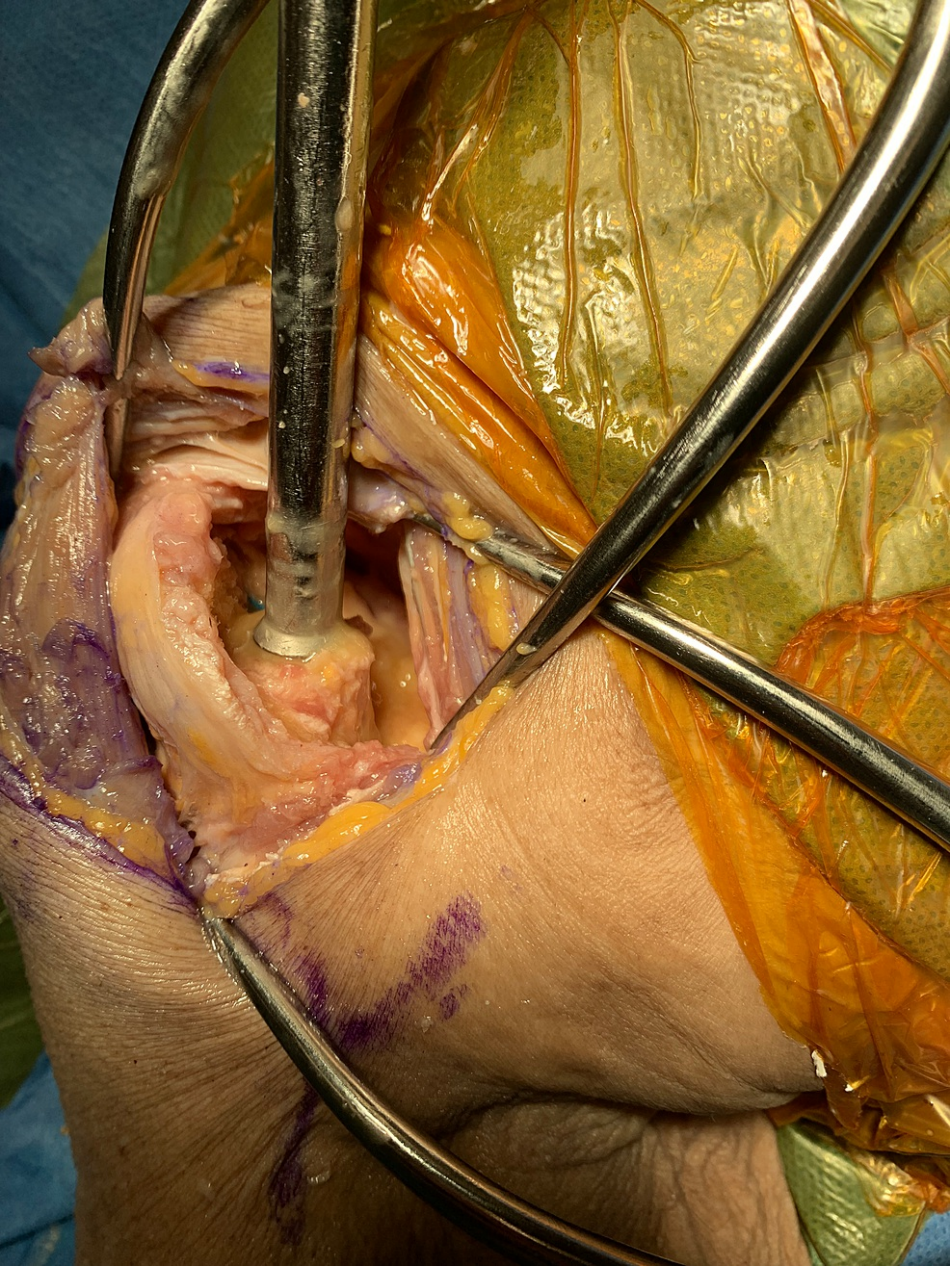
Corkscrew within the canal of the fibular strut.

A handheld corkscrew from the standard hip hemi-arthroplasty set is used (Figure 5). The corkscrew should be placed centrally into the canal of the fibula strut and carefully advanced down the canal of the strut. Once there is good purchase, which is tested with a small tug, the strut is removed by simply pulling the graft up and free of the canal (Figure 6).

Gently curette the canal after the strut is removed. Although removing the fibular strut from the humeral canal is a key component for the shoulder arthroplasty, there is often a bony void left after removal of the strut. If the bony quality is deemed to be poor prior to removing the strut, we recommend leaving the strut in place while preparing the glenoid and then coming back to the humerus to determine if the strut should or should not be removed for placement of the humeral component

After the graft was removed the humeral canal was curetted out and found to be devoid of iatrogenic fracture. Next, the glenoid and humerus were prepped appropriately, and an Arthrex (Naples, FL, USA) RTSA was inserted uneventfully. 

## Discussion

The definitive management of proximal humerus fractures is a highly debated topic within the orthopedic community. Multiple factors must be considered when choosing which option will provide the best functional outcome for the patient. Fracture pattern, integrity of the rotator cuff, prior osteoarthritis of the shoulder, underlying medical conditions and overall functional baseline of the patient are considered. Non-operative management is a widely accepted treatment option for one- or two-part patterns [27,28]. However, for the treatment of three- and four-part proximal humerus fractures there are multiple options and even in the most expert hands, choosing the correct option is difficult. The options most supported within the literature are locked lateral plating, intramedullary nail, hemiarthroplasty, and reverse total shoulder arthroplasty [29-38].

The PROFHER study evaluated the clinical effectiveness of surgical versus nonsurgical management of displaced proximal humerus fractures involving the surgical neck. The results of this study showed no significant difference between surgical (plate and screws or hemiarthroplasty) and non-surgical management [27]. This landmark study demonstrated non-surgical management is a reasonable option for simple two-part fracture patterns. However, in elderly patients with three- or four-part proximal humerus fractures the options become more limited and the current trend is acutely replacing rather than attempting to fix these fractures. The DelPhi study demonstrated a significant advantage at two-year follow-up in favor of RTSA over ORIF for the treatment of displaced OTA/AO type-B2 and C2 proximal humerus fractures in the elderly population [28]. Implementing RTSA primarily for proximal humerus fractures is far more straightforward than the revision setting. Kuhlman et al. demonstrated similar five-year outcomes with performing RTSA for acute proximal humerus fractures versus delayed treatment [34]. RTSA has gained popularity for the acute treatment of proximal humerus fractures due to the complications associated with locked plate and screws when used for ORIF.

The most common complications after ORIF with locked plate and screws are screw cutout, malreduction, malunion, non-union, avascular necrosis and infection [5-10]. Complication rates between 20-40% have been reported and with up to 25% of patients requiring revision surgery [6]. One study reviewing complications after ORIF found that 57% of complications were due to screw cut-out which led to glenoid destruction in 33% of patients [6]. This high complication rate has led surgeons to seek additional augmentation options during ORIF of proximal humerus. Fibular strut endosteal augmentation has been described in studies to provide additional support and decrease implant failure rates in displaced fractures with varus coronal malalignment that have significant metaphyseal bone loss with or without medial calcar comminution [11,12]. Use of fibular strut augmentation with lateral locking plates has shown to aid in preventing varus collapse, decrease rate of screw cut-out, improve healing rates, reduce avascular necrosis of the humeral head and maintain overall stability of the lateral locking plate leading to improved functional and radiographic outcomes [11-17]. Although the rates of complication and failure are relatively low, revision after fibular strut augmentation poses a unique and technically challenging problem.

There is paucity within the literature about removal of a fibular strut allograft for conversion from locked plate and screws to shoulder arthroplasty. One technique that has been described is using a Midas burr to remove the fibular-humerus interface, followed by use of osteotomes or a Cobb elevator to loosen the strut and then pull it out [18]. Although this is a simple technique, it risks iatrogenic humeral shaft fracture, fragmentation of the strut and loss of humeral bone stock. Another technique described uses a tenodesis reaming set to ream out the fibular strut, but again, this technique risks loss of bone stock and iatrogenic humeral shaft fracture [19].

## Conclusions

The technique being described in this paper simply requires a handheld corkscrew commonly utilized in hip arthroplasty. The advantages compared to others are: this technique does not require opening new trays, theoretically decreases risk of iatrogenic fracture, and essentially creates no loss of humeral bone stock beyond the disruption of the fibula-humeral interface. It is the opinion of these authors that this novel technique is reproducible and can be utilized safely and simply for the removal of fibular strut allografts in the setting of revision shoulder arthroplasty.
